# Broad Luminescence Generated by IR Laser Excitation from CsPbBr_3_:Yb^3+^ Perovskite Ceramics

**DOI:** 10.3390/molecules28145324

**Published:** 2023-07-11

**Authors:** Mariusz Stefanski, João Marcos Gonçalves, Wieslaw Strek

**Affiliations:** Institute of Low Temperature and Structure Research, Polish Academy of Sciences, 50-422 Wroclaw, Poland

**Keywords:** inorganic bromide perovskite, broadband laser induces emission, threshold behavior

## Abstract

This paper demonstrates the generation of broadband emission in the visible and infrared ranges induced by a concentrated beam of infrared radiation from CsPbBr_3_ ceramics doped with Yb^3+^ ions. The sample was obtained by the conventional solid-state reaction method, and XRD measurements confirmed the phase purity of the material crystallizing in the orthorhombic system. Spectroscopic measurements required further sample preparation in the form of ceramics using a high-pressure press. The research showed that as the excitation power increases, the emission intensity does not increase linearly from the beginning of the experiment. Irradiation of the material results in the accumulation of the delivered energy. Absorption of a sufficient number of photons triggers avalanche emission. It was found that the most intense luminescence is produced in a vacuum. Changes in conductivity were also observed, where the excitation was able to lower the resistivity of the material and it was highly dependent on the excitation power. The mechanism responsible for the generation of the observed phenomenon involving intervalence charge transfer (IVCT) transitions has been postulated.

## 1. Introduction

Interest in lead halide perovskites has been increasing in recent years due to their unique optical properties, such as precise control of absorption and emission color, either by size or composition. The name “perovskite” was first given by Gustav Rose in 1839 after Russian mineralogist Lev Perovski to the mineral CaTiO_3_. However, it is now used as a general term for several materials that present the general formula APbX_3_, where A can be either an organic cation (such as formamidinium or methylammonium) or an inorganic cation (such as Cs^+^, which is the most common) exhibiting a network of corner-shared PbX_6_ octahedra, and crystalizing in either cubic, orthorhombic, or tetragonal phases [1], while X can be substituted by oxygen or halide elements, such as Br. The attention to this type of materials has resulted in a number of articles in recent years indicating the application of perovskites, in particular as potential candidates for photovoltaic devices, for example [2,3,4,5,6,7,8,9].

When submitted to infrared laser excitation passing through a focusing lens, a wide variety of materials present a bright broadband emission, commonly called laser-induced emission (LIE). One of the first reports of this phenomenon was made in 2010 by Wang and Tanner, where an intense white light generation was achieved in Ln_2_O_3_ (where Ln was Sm^3+^, Tm^3+^, and Yb^3+^) and CeO_2_ [10]. This emission has a few particular characteristics, such as exclusively appearing with focused excitation, a non-linear dependence of its intensity as a function of excitation power, and pressure surrounding the material, to cite a few. More recently, changes in resistivity were also investigated in some materials and it was found that, upon focused infrared excitation, a decrease in resistivity was observed, coupled with the simultaneous generation of current with LIE, which indicates that the phenomenon is related to promotion of electrons, i.e., it has an electronic nature. It is worth highlighting that this change was strongly dependent on excitation power, meaning that, with higher power, there was a more pronounced decrease in resistivity. This was observed in several materials, such as La_1-x_Nd_x_AlO_3_ [11], GaN [12], LiYbP_4_O_12_ [13], Sr_2_CeO_4_ [14], and graphene [15], to cite a few.

Since its first description, a wide variety of materials were described as capable of producing LIE, such as transition metal- or lanthanide-doped garnets [16,17], transparent ceramics [18,19], a wide variety of inorganic nanocrystals, such as Sr_2_CeO_4_ [14,20,21], or LnAlO_3_ (where Ln can be different lanthanides) [11,22], Sn-based clusters [23,24], YVO_4_ [25], silica porous materials [26], and even carbon-based materials, such as graphene [27] or diamonds [28]. Despite the fact that many materials have been discovered for LIE, the mechanism is still not agreed upon, and several have been proposed, such as blackbody emission [29,30], oxygen vacancies [25], photon avalanche [31,32], and radiative energy loss (*bremsstrahlung* emission) [24], to cite a few. However, multiphoton ionization and subsequent intervalence charge transfer (IVCT) have been receiving significant attention. In this mechanism, the absorption of multiple photons leads to the ionization of the material following Keldysh’s theory [33], subsequently creating ionic pairs that undergo an IVCT. To further support the IVCT model, the presence of Yb^2+^ ions after laser excitation has been observed in Yb^3+^-doped YAG [34]. Furthermore, the competing nature of LIE and the MLCT (metal-ligand charge transfer) emission band in Sr_2_CeO_4_ [20], and, more recently, the threshold dependence on relative permittivity of LIE in alcohols [35], have been reported, which are compelling evidence of the involvement of ionization and a possible IVCT in this complex emission phenomenon. Furthermore, changes in the resistance of the material upon focused laser excitation accompanied by the emission seem to indicate a close relationship between light emission and the ejection of electrons [15,35].

Two main applications were linked to LIE: since it presents a high color rendering index (CRI) close to 100 [36,37,38], it is very favorable for indoor lighting applications. Furthermore, the ejection of electrons was recently linked to the production of H_2_ [39,40,41], adding great value for research in laser-induced emission since it is a very active field of research for green energy generation. Additionally, LIE is capable of increasing the light power output in tungsten lamps [42], which could significantly increase interest in it.

Although a high number of materials were described to produce LIE, lead bromide perovskites have not yet been explored in this manner. Due to the surging interest in this material, a new possibility for applications might be achieved with LIE. Therefore, here we investigate the emission properties of CsPbBr_3_:10%Yb^3+^ under focused infrared excitation. The characteristics of LIE were identified and described, coupled with an IVCT mechanism proposition.

## 2. Results and Discussion

### 2.1. Structural and Optical Properties

**Figure 1:**
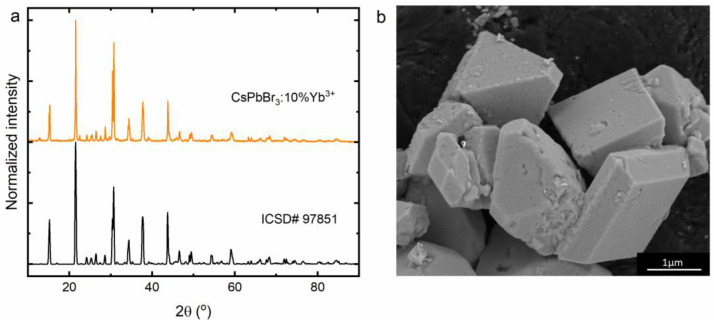
X-ray diffraction pattern (**a**) and SEM image (**b**) of the CsPbBr_3_:10%Yb^3+^ perovskite powder.

To confirm the phase purity of the CsPbBr_3_:10%Yb^3+^ perovskite powder, XRD measurements were collected and are illustrated in Figure 1a. It can be observed that the obtained result is consistent with the ICSD# 97851 standard, which means that the investigated material crystallizes in an orthorhombic crystallographic system in *Pbnm* symmetry. Our report shows that using the dry chemistry method in the form of a solid-state reaction leads to obtaining a material that is characterized by micrometric average grain sizes (Figure 1b). Moreover, the resulting crystallites show irregular geometric shapes and their walls are very smooth. Aggregation of CsPbBr_3_:Yb^3+^ crystallites was not observed. It is worth emphasizing that, according to the literature reports, dopant ions substitute lead positions due to the similarity of their ionic radii [43,44]. Nevertheless, the introduction into the structure of a dopant with a smaller ionic radius compared to that of the substituted ion results in the formation of defects (probably lead vacancies—V_Pb_ [45]) to compensate for the difference in charge.

**Figure 2:**
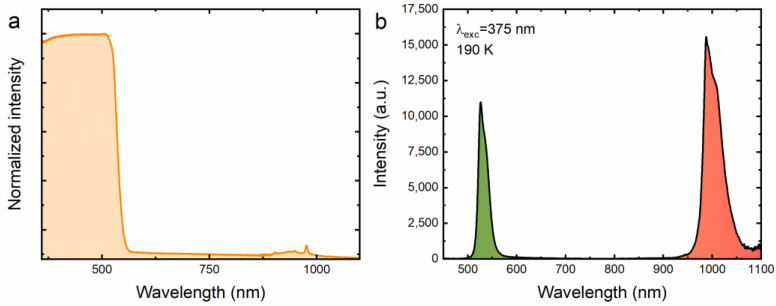
Absorption (**a**) and emission (**b**) spectra of the CsPbBr_3_:10%Yb^3+^ perovskite powder recorded at 300 K and 190 K, respectively.

In order to prove that Yb^3+^ ions have embedded themselves in the crystal structure of the investigated material, absorption and emission spectra of the CsPbBr_3_:Yb^3+^ perovskite powder were recorded upon excitation with a laser diode operating in the 375 nm range measured at 300 K and 190 K, respectively. In Figure 2a, it is clear that from the ultraviolet to the greenish range the intense host band dominates [46]. In addition, a weaker absorption band in the near-infrared range can be found, which is typical of ytterbium ions exhibiting a +3 oxidation state, representing the transition between the ^2^F_7/2_ ground state and the ^2^F_5/2_ excited state [47]. It was found that excitation of the material in the UV range leads to energy splitting between two emission centers, as presented in Figure 2b. The first is associated with exciton luminescence, while the second is related to the emission of Yb^3+^ ions, manifested by an intense band with a maximum at 530 nm and 990 nm, respectively [48]. The detection of a strong emission band in the NIR range unequivocally confirms the presence of Yb^3+^ ions in the studied material. The exclusion of the formation of Yb^2+^ ions, which theoretically should more easily locate in the positions of Pb^2+^ ions due to their similar charge, means that before irradiation of the sample with a concentrated beam of a near-infrared laser diode, there are no such ions in the CsPbBr_3_:Yb^3+^ structure. The obtained result is extremely important for explaining the mechanism of anti-Stokes luminescence generated from CsPbBr_3_:Yb^3+^ ceramics, which is described later in this article.

**Figure 3:**
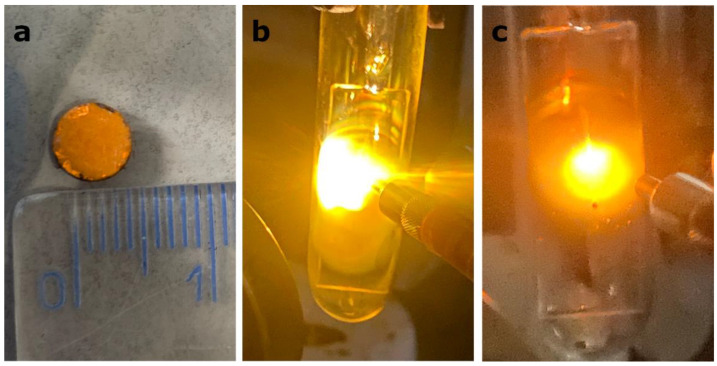
Digital photograph of the CsPbBr_3_:10%Yb^3+^ ceramics in daylight (**a**), under focused 808 nm excitation (**b**) and under focused 975 nm excitation (**c**). The former two are included to showcase the visual characteristics of LIE.

Because LIE generation requires good contact between grains, the irradiation of powder with a concentrated infrared laser beam results in a lack of broadband luminescence. Therefore, ceramics of CsPbBr_3_:10%Yb^3+^ with a 0.5 cm diameter were prepared, as shown in Figure 3a. When these ceramics were exposed to a focused infrared laser excitation, a clear bright laser-induced emission was observed (Figure 3b,c). Regardless of whether the excitation wavelength was 808 nm or 975 nm, the characteristics of the emission were very similar, namely a warm light in the orange region, characteristic of several other materials presenting LIE [11,22,36,37]. The precise characterization, i.e., CIE coordinates and CRI, will be explored later.

**Figure 4:**
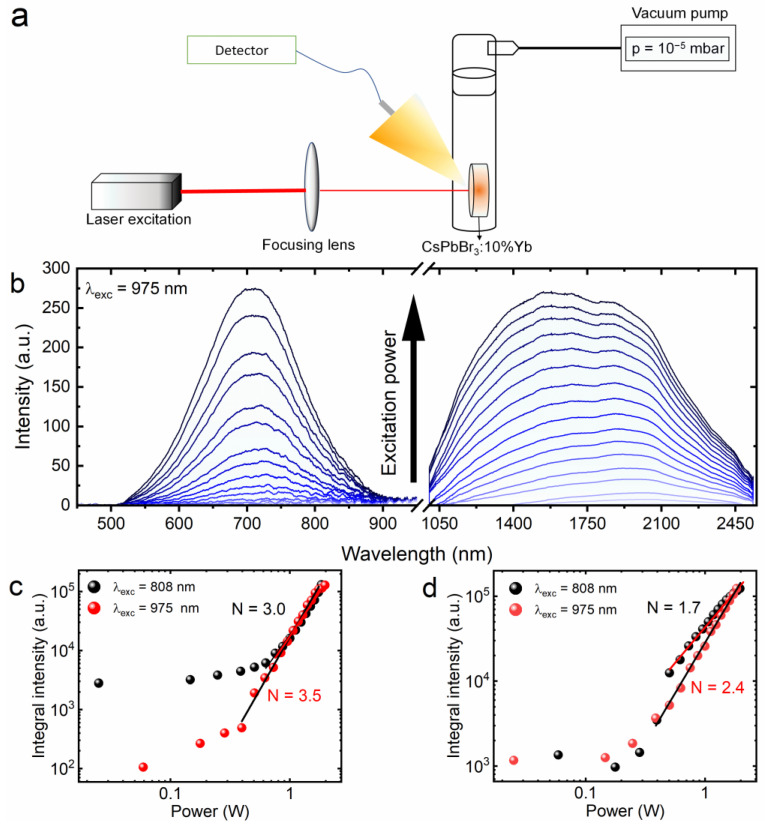
Schematic representation of the experimental setup used for power dependence measurements (**a**). Laser-induced emission spectra of CsPbBr_3_:10%Yb^3+^ under focused 975 nm excitation (**b**). Intensity dependence on excitation power under focused 808 nm and 975 nm for the visible range (**c**) and infrared range (**d**).

The spectroscopic characterization of the effect observed from the CsPbBr_3_:10%Yb^3+^ ceramics was based on the registration of the emission power dependence in two different experiments using two different wavelengths of infrared radiation according to the diagram presented in Figure 4a. It is worth noting that the experiments were performed in a homemade quartz chamber, inside of which a high vacuum (10^−5^ mbar) was established using a turbomolecular pump. Moreover, to achieve broadband emission, focusing the excitation beam with a lens was essential. The luminescence was observed in both the visible and infrared spectral ranges (Figure 4b), but the need to use two different detectors led to the impression that two bands were generated when, in fact, it was one broad emission band. Figure 4c,d clearly show that for low values of excitation power, the broadband emission was not observed, regardless of the excitation wavelength. A drastic increase in luminescence intensity occurred no sooner than above 0.4 W of laser diode power. This phenomenon is called photon avalanche emission, which empirically can be represented by the power law $I\propto P^{N}$, where *I* is the LIE intensity, *P* stands for the power of the excitation source and the *N* parameter means the number of absorbed photons involved in the process [26,49]. Such threshold behavior is characteristic of the generation of broadband emission that is also observed in other materials [19,50,51]. Although two different excitation sources were used, the order of magnitude of the received *N* parameter is similar between them. However, it can be seen that as the excitation energy of the laser diode increases, the *N* parameter decreases. Reports already published in the literature considering the generation of broadband emission show that this is a common effect [12,21]. Surprisingly, the magnitude of the *N* parameter varies depending on the measured spectral range. Usually, smaller values are determined for the NIR region, but the trend remains the same as that for the VIS region [12,27]. In order to keep the article clear, and because of the similarity of the obtained spectra, only the emission recorded at 975 nm is presented. 

**Figure 5:**
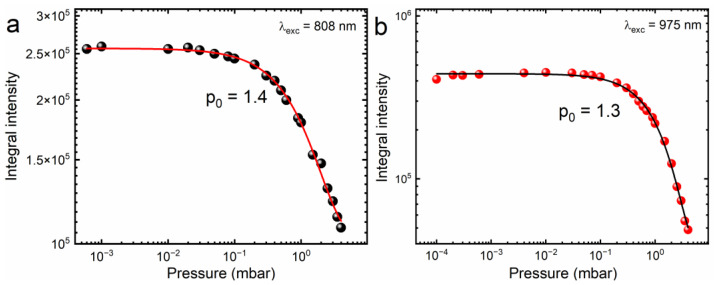
Integral intensity dependence on ambient pressure for focused 808 nm excitation (**a**) and 975 nm (**b**) for CsPbBr_3_:10%Yb^3+^ ceramics.

Figure 5 shows the relationship between the pressure surrounding the CsPbBr_3_:10%Yb^3+^ ceramics and the intensity of the broadband luminescence upon excitation of the studied material with focused light provided by a near-infrared laser diode. It can be observed that the LIE is most efficient in the pressure range from 10^−5^ mbar to 0.1 mbar. For higher pressures, the broadband emission intensity degrades quickly and then its quenching occurs. The nature of this phenomenon can be explained by a heat dissipation model described empirically by the following formula:

(1) $$I_{em}=I_{0}exp\left(-\frac{p}{p_{0}}\right)$$

where *p*_0_ is the critical magnitude of pressure of the surrounding atmosphere above which the LIE intensity begins to decrease and *I*_0_ is the initial intensity. Briefly, this model assumes that the temperature of the sample is reduced at atmospheric pressure when the investigated material is irradiated with IR laser diode light, either due to the scattering of the excitation beam by air molecules, or thermal diffusion between the surface of the sample and air molecules surrounding the material, where part of the energy is lost to heat instead of emitting light. Consequently, quenching of the luminescence is observed. Similar behavior has been reported in the literature, not only in the air atmosphere but also in the environment of other inert gases [10]. It is worth mentioning that, contrary to other materials, the emission in ambient pressure was not completely quenched, showcasing the possibility of exploring LIE to produce H_2_, as explored previously in aluminate perovskites and graphene by our group [39,52,53]; however, stability of this material in solvents is a great concern, since water is known to decompose the material, for example.

**Figure 6:**
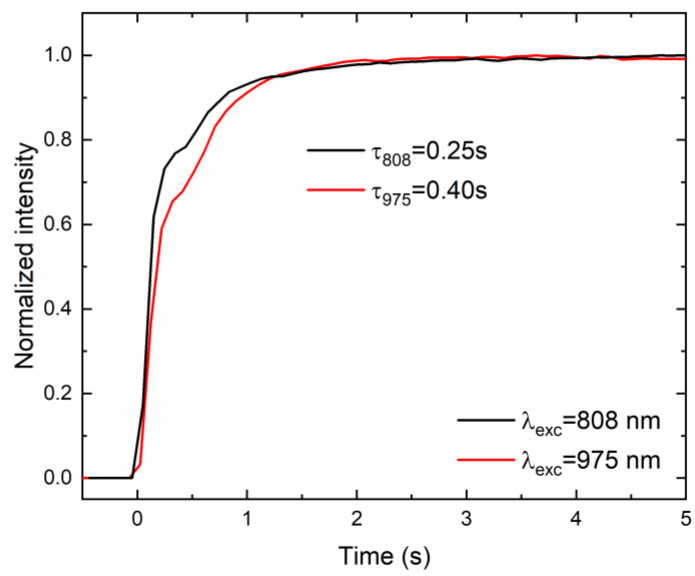
The rise time of the LIE from CsPbBr_3_:10%Yb^3+^ ceramics under 808 nm and 975 nm using fixed 1.0 W excitation power.

Since the proposed technique for the generation of broadband emission from CsPbBr_3_:10%Yb^3+^ ceramics is unusual and pulsed excitation is insufficient to achieve it, conventional luminescence decays could not be recorded. However, in order to obtain deeper insights into the nature of the observed phenomenon, luminescence rise times were recorded upon both excitation lines by measuring the time required to reach the maximum emission intensity from the activation of the excitation source (see Figure 6). It was found that the aforementioned time is relatively long and prolonged as the excitation energy increases from 0.25 s to 0.40 s for 808 nm and 975 nm excitation lines, respectively. This behavior can be attributed to the time required to shift an electron from the valence band to the conduction band. In this case, higher excitation energy accelerates the processes that occur; in other words, the higher the excitation energy, the shorter the mentioned time [11,20].

**Figure 7:**
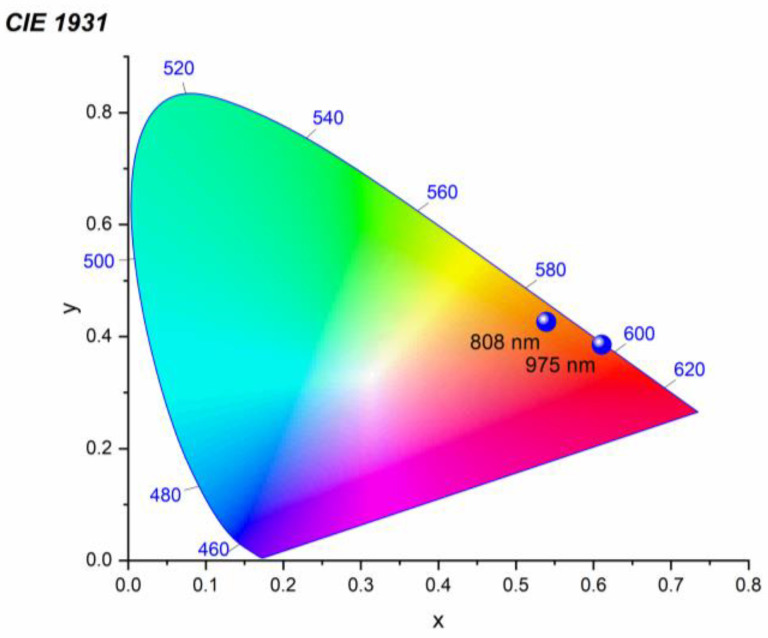
CIE chromaticity diagram of the LIE produced under focused 808 nm and 975 nm excitations for the CsPbBr_3_:10%Yb^3+^ ceramics. The spectra with maximum intensity were chosen to construct the diagram.

In order to precisely characterize the emission observed from CsPbBr_3_:Yb^3+^ ceramics presented in Figure 3b,c, its chromatic coordinates were determined based on the photoluminescence spectrum showing the highest emission intensity. The result of the calculation can be found in Figure 7. It was found that the technique proposed for producing broadband emission from the investigated material leads to the acquisition of luminescence exhibiting (x = 0.53908, y = 0.42632), (x = 0.61055, y = 0.38551) coordinates at 808 nm and 975 nm excitations, respectively. As a result, the observed emission is located in the orange-reddish region in the CIE diagram. Further analysis showed that the obtained luminescence reveals a warm color in the range of 2000 K and 1300 K, and has a color rendering index (CRI) equal to 73 and 83, at 808 nm and 975 nm excitations, respectively.

### 2.2. Photoconductivity

**Figure 8:**
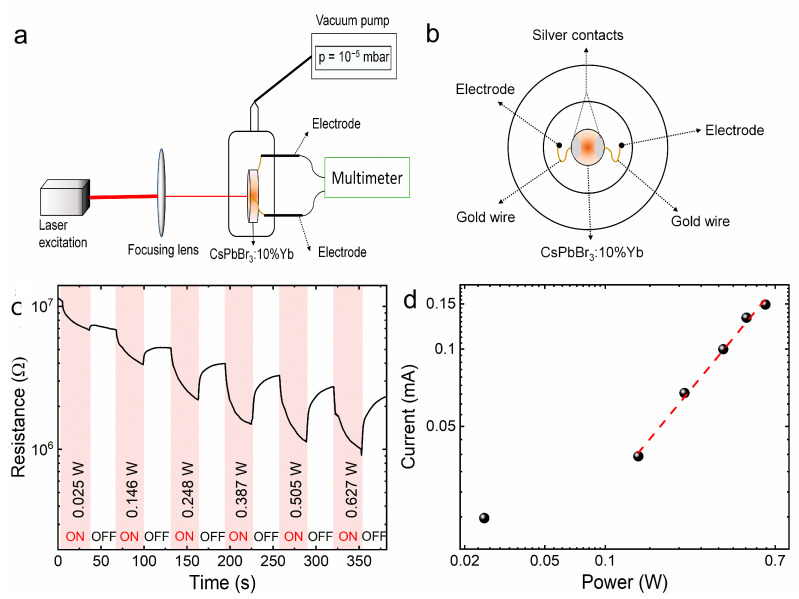
Schematic representation of the homemade experimental setup for photoconductivity measurements: side view (**a**) and front view (**b**). Resistance changes of the CsPbBr_3_:10%Yb^3+^ ceramics under on–off cycles with 30 s of focused 975 nm excitation (**c**). Generated current as a function of excitation power (**d**).

Photoconductivity measurements were carried out on a homemade system comprising a quartz cell with electrodes. Gold wires were attached to the surface of the ceramic with silver contacts and connected to the electrodes, and the measurements were made in a vacuum (10^−5^ mbar). The experimental apparatus is illustrated in Figure 8a,b. Ejection of electrons in LIE was observed in several materials by means of changes in resistance with focused excitation. For CsPbBr_3_:10%Yb^3+^ ceramics, a similar behavior was observed, as can be seen in Figure 8c. The experiment was performed with on–off cycles with a 30 s duration, in which the excitation power was increased after every cycle. Two observations can be clearly made: first, when the excitation was turned on, a decrease in resistance occurred, indicating the promotion of electrons to the conduction band. This decrease can also be divided into two different steps, an initial fast one due to the ionization of the sample, and a slower component due to heating [54].

Furthermore, the magnitude of the decrease is highly dependent on excitation power, indicating that a higher power induces a more pronounced ionization on the material. Similar behavior was observed in LaAlO_3_:Nd^3+^ and Y_2_Si_2_O_7_ [11,55], to cite a few. Additionally, a photocurrent was also detected, which presented a non-linear behavior (Figure 8d). Both these facts indicate an ionization process that will be important to the proposed mechanism.

### 2.3. Energy Transfer Mechanism

Considering the mechanism that may be responsible for the laser-induced emission (LIE) observed from CsPbBr_3_ ceramics doped with Yb^3+^ ions, one very important factor must be taken into account, namely the temperature of the sample during exposure to the concentrated infrared beam. Previous literature reports have proven that the temperature of the studied material is relatively low and is usually no higher than 1000 °C [10,56]. This means that the blackbody theory, which explains well the shape of the emission band and the pressure dependence but does not explain the threshold nature of the observed phenomenon, cannot be applied here. In addition, the observation of LIE in a liquid N_2_ temperature also contradicts the blackbody emission [57]. Additional confirmation of this fact is the lack of change in the position of the luminescence maximum during the measurements of emission intensity as a function of the near-infrared laser diode power density. Moreover, the recent discovery sheds new light on the studied broadband emission. It is worth mentioning that the LIE generated in the presented manner is a surface phenomenon and occurs only from the point of exposure to the laser beam, and not from the entire surface of the material [18]. Furthermore, it was found that the LIE is accompanied by the emission of hot electrons, which is of particular importance for using the studied phenomenon in the photocatalysis process for hydrogen production [53], thereby expanding the possibility for application of this phenomenon. Taking into account all the mechanisms of LIE generation proposed in the literature so far, the most likely seems to be the one associated with the formation of [Yb^3+^-Yb^2+^] ion pairs due to the illumination of the sample with focused IR radiation leading to multiphoton absorption and ionization of microcrystalline CsPbBr_3_:Yb^3+^ perovskite ceramics. As a result, electron promotion from the valence to the conduction band occurs. The free electrons then combine with Yb^3+^ to form Yb^2+^ ions and a charge transfer transition between the mixed valence pairs (IVCT) arises manifested by broadband emission. It is worth noting that this hypothesis was already supported by ab initio calculations [58,59]. The previously mentioned surface defects in the form of lead vacancies may also participate in the proposed mechanism. However, further detailed studies are needed to clarify this.

## 3. Materials and Methods

CsPb_0.9_Br_3_ perovskite powder of micrometric size doped with 10% of Yb^3+^ ions was prepared by the traditional solid-state reaction method. For this purpose, stoichiometric amounts of starting materials such as CsBr (99.9%, Sigma-Aldrich, Saint Louis, Missouri, USA), PbBr_2_ (≥98%, Sigma-Aldrich), and YbBr_3_·xH_2_O (99.9%, Alfa Aesar, Haverhill, MA, USA) were weighed and ground intensively in an agate mortar in the presence of ethylene alcohol. Such an environment was required to ensure better mixing of the reactants, which was carried out until the alcohol evaporated completely. The powder prepared in this way was placed in a quartz boat and then heated in a tube furnace in an inert atmosphere (nitrogen) under the following regime: 550 °C/3 h, 480 °C/48 h, and 300 °C/24 h. In order to provide better contact between the grains, the resulting orange powder was sintered into ceramics on a press using the low-temperature high-pressure (LTHP) technique at 500 °C under 8 GPa. It is worth emphasizing that the orange color of the sample is a characteristic of the studied material and not the result of doping with lanthanide.

The X-ray diffraction (XRD) pattern was measured with an X’Pert PRO powder diffractometer (Malvern Panalytical, Malvern, UK) equipped with a linear PIXcel detector and using Cu Kα radiation (λ = 1.54056 Å). The chemical composition and general morphology of the samples were checked using a FE-SEM microscope (NanoSEM 230, FEI Nova, Hillsboro, OR, USA). The absorption spectra were measured in the back scattering mode using an Agilent Cary 5000 spectrophotometer (Agilent Technologies, Santa Clara, CA, USA). The Stokes emission spectrum was recorded using a Hamamatsu photonic multichannel analyzer PMA-12 equipped with a BT-CCD linear image sensor (Hamamatsu Photonics, Iwata, Japan). A laser diode operating under a 375 nm excitation line was applied as the excitation source. The temperature of the samples during emission measurements was controlled by Linkam THMS600 Heating/Freezing Stage (Linkam Scientific Instruments Ltd., Salfords, United Kingdom). The anti-Stokes emission spectra were measured using focused CW laser diodes (808 and 975 nm) as excitation sources and an AVS-USB2000 Spectrometer (Avantes, Apeldoorn, The Netherlands) as a detector for the visible range and Ocean Optics Nirquest 512-2.5 (Ocean Optics, Inc., Dunedin, FL, USA) as a detector for the near-infrared range. All of the emission spectra were corrected for the detector sensitivity. The rise times of the emission intensity were recorded using a PM101U power meter equipped with a S122C head (Thorlabs, Newton, NJ, USA). Photocurrent measurements were performed using a Keithley 2410 SourceMeter (Keithley Instruments, Cleveland, OH, USA) at room temperature under 975 nm CW laser diode excitation, with 150 V of bias voltage. For electrical measurements, silver electrodes with a gold wire were attached to the ceramic. The sample was illuminated with a focused beam of a 975 nm laser diode between the electrodes. The measurements were performed in 30 s cycles during which the laser diode was turned on and off, varying its power in the range of 0–0.6 W. All measurements were performed at low-pressure conditions using a vacuum cell supplied with a Turbomolecular Drag Pump TMH071 P and a TC 600 electronic drive unit (Pfeiffer, Aßlar, Germany). The focusing lens had a focal length of 40 mm; therefore, the sample was positioned at a distance of 4 cm from it to obtain the smallest excitation area achievable.

## 4. Conclusions

The conducted research demonstrated that the dry chemistry method, namely the solid-state reaction, can be successfully used to synthesize micrometric inorganic bromide-based perovskites doped with Yb^3+^ ions in powder form. The obtained material after pressure treatment served as a target illuminated by a concentrated infrared beam, which responded with broadband emission reaching far beyond the visible range. The luminescence exhibited the highest intensity in a vacuum and strong nonlinear power dependence of the NIR excitation laser. It was characterized by a pleasant color with a high CRI. Upon focused laser excitation, a decrease in resistivity, as well as the appearance of current, were observed, indicating both photoionization and heating of the material; the latter was due to the high power density of the excitation achieved with focusing. Interestingly, both the resistivity decrease and the current were strongly dependent on excitation power, suggesting that higher excitation power induces higher degrees of ionization. The mechanism behind the observed phenomenon is probably related to the [Yb^2+^-Yb^3+^] ion pairs induced by the laser beam during energy absorption and the following radiative depopulation through intervalence charge transfer transitions. Moreover, Stokes absorption and emission measurements showed the presence of Yb^3+^ ions and the absence of a trace of Yb^2+^ in the studied material, indicating that ytterbium(II) ions appear as a result of irradiation of the CsPbBr_3_:Yb^3+^ ceramics with a concentrated infrared laser beam. Due to the high CRI and warm light emission, the LIE generated from this material can be used for indoor lighting, and due to the ejected electrons in ambient pressure, it might be even capable of producing H_2_ in solvents such as alcohols.

## Data Availability

The data presented in this study are openly available in Zenodo at DOI: 10.5281/zenodo.8006575.

## Sample Availability

Samples of the compounds are not available from the authors upon request.
